# Supplementary material for: Design of immunogens for eliciting antibody responses that may protect against SARS-CoV-2 variants
Source: PLoS Comput Biol. 2022 Sep 26;18(9):e1010563. doi: 10.1371/journal.pcbi.1010563 (PMC9536555; doi:10.1371/journal.pcbi.1010563)
Supplement: S1 Table — (DOCX) [file pcbi.1010563.s001.docx]

**S1 Table**. Coronaviruses used in conservation analysis along with the PDB ID of the spike protein, the genus of the coronavirus, and the coronavirus's host receptor.

| Coronavirus Name | PDB ID | Genus | Receptor |
| --- | --- | --- | --- |
| SARS-CoV-2 | 6VXX | Beta | ACE2 (1) |
| SARS-CoV | 5X58 | Beta | ACE2 (2) |
| MERS | 5X59 | Beta | DPP4 (3) |
| 229E | 6U7H | Alpha | APN (4) |
| HKU1 | 5I08 | Beta | Sialic acid (5) |
| OC43 | 6NZK | Beta | Sialic acid (5) |
| NL63 | 7KIP | Alpha | ACE2 (6) |
| IBV | 6CV0 | Gamma | Sialic acid (7) |
| MHV | 3JCL | Beta | CEACAM1 (8) |
| FIPV | 6JX7 | Alpha | APN (9) |
| SADS | 6M16 | Alpha | Unknown |
| HKU2 | 6M15 | Alpha | Unknown |
