# Supplementary material for: Design of immunogens for eliciting antibody responses that may protect against SARS-CoV-2 variants
Source: PLoS Comput Biol. 2022 Sep 26;18(9):e1010563. doi: 10.1371/journal.pcbi.1010563 (PMC9536555; doi:10.1371/journal.pcbi.1010563)
Supplement: S2 Table — (DOCX) [file pcbi.1010563.s002.docx]

**S2 Table.** Class 1 and class 2 antibodies used to identify RBD escape mutations.

| Antibody Name | Class |
| --- | --- |
| C105 | 1 (10) |
| COV2-2165 | 1 (11) |
| COV2-2196 | 1 (12) |
| COV2-2832 | 1 (11) |
| LY-CoV016 | 1 (13) |
| REGN10933 | 1 (13) |
| S2E12 | 1 (14) |
| S2H14 | 1 (14) |
| C002 | 2 (10) |
| C121 | 2 (10) |
| C144 | 2 (10) |
| COV2-2050 | 2 (11) |
| COV2-2096 | 2 (11) |
| COV2-2479 | 2 (11) |
| LY-CoV555 | 2 (15) |
| S2D106 | 2 (14) |
| S2H13 | 2 (14) |
| S2H58 | 2 (14) |
| S2X16 | 2 (14) |
| S2X58 | 2 (14) |
