# Supplementary material for: Design of immunogens for eliciting antibody responses that may protect against SARS-CoV-2 variants
Source: PLoS Comput Biol. 2022 Sep 26;18(9):e1010563. doi: 10.1371/journal.pcbi.1010563 (PMC9536555; doi:10.1371/journal.pcbi.1010563)
Supplement: S3 Table — Numbering is based on the SARS-CoV-2 spike protein (PDB ID: 6VXX). (DOCX) [file pcbi.1010563.s003.docx]

**S3 Table.** Residues making up the conserved region in the spike S2 domain. Numbering is based on the SARS-CoV-2 spike protein (PDB ID: 6VXX).

| Residues |  |  |  |  |
| --- | --- | --- | --- | --- |
| 719 | 769 | 861 | 923 | 955 |
| 722 | 770 | 862 | 924 | 956 |
| 723 | 771 | 863 | 925 | 957 |
| 724 | 772 | 864 | 926 | 958 |
| 727 | 775 | 865 | 927 | 959 |
| 730 | 781 | 869 | 928 |  |
| 731 | 782 | 871 | 930 |  |
| 733 | 802 | 873 | 931 |  |
| 735 | 805 | 874 | 932 |  |
| 736 | 816 | 877 | 934 |  |
| 737 | 817 | 878 | 935 |  |
| 738 | 818 | 879 | 945 |  |
| 741 | 819 | 898 | 947 |  |
| 742 | 820 | 899 | 948 |  |
| 749 | 821 | 901 | 949 |  |
| 759 | 822 | 902 | 950 |  |
| 762 | 823 | 905 | 951 |  |
| 763 | 826 | 906 | 952 |  |
| 766 | 857 | 907 | 953 |  |
| 767 | 860 | 920 | 954 |  |
