# Supplementary material for: Design of immunogens for eliciting antibody responses that may protect against SARS-CoV-2 variants
Source: PLoS Comput Biol. 2022 Sep 26;18(9):e1010563. doi: 10.1371/journal.pcbi.1010563 (PMC9536555; doi:10.1371/journal.pcbi.1010563)
Supplement: S1 Text — (DOCX) [file pcbi.1010563.s004.docx]

## S1 Text. Collection and processing of NCBI coronavirus sequences

Coronavirus spike protein sequences were obtained by searching for the coronavirus name in the NCBI Protein database and downloading spike protein sequences. Sequences uploaded as of May 20, 2021 were obtained as this was when the download was done. Then, sequences not corresponding to the spike protein of the appropriate coronavirus were manually removed based on the organism and protein name, leading to ~4000 coronavirus sequences in total. The sequences of a particular coronavirus were aligned to the sequence of the spike protein PDB structure (PDB structure code is in Table S1) using the ClustalW algorithm (1) in MEGAX (2) with gap opening penalty of 10 and gap extension penalty of 0.2 (sequences available at <https://github.com/ericzwang/sars2-vaccine/blob/main/data/aligned-cov-sequences.gz>).

References

1. Thompson JD, Higgins DG, Gibson TJ. CLUSTAL W: improving the sensitivity of progressive multiple sequence alignment through sequence weighting, position-specific gap penalties and weight matrix choice. Nucleic Acids Research. 1994;22(22):4673-80.

2. Kumar S, Stecher G, Li M, Knyaz C, Tamura K. MEGA X: Molecular Evolutionary Genetics Analysis across Computing Platforms. Molecular Biology and Evolution. 2018;35(6):1547-9.
