# Supplementary material for: Design of immunogens for eliciting antibody responses that may protect against SARS-CoV-2 variants
Source: PLoS Comput Biol. 2022 Sep 26;18(9):e1010563. doi: 10.1371/journal.pcbi.1010563 (PMC9536555; doi:10.1371/journal.pcbi.1010563)
Supplement: S2 Text — (DOCX) [file pcbi.1010563.s005.docx]

## S2 Text. Estimation of antibody footprint area

Antibody footprint area was estimated using the structures of ten antibodies complexed with spike proteins (PDB IDs: 6W41, 7L7D, 7KMG, 7R7N, 7R6W, 7JX3, 7M7W, 6WPS, 7K8Z, 7K90). For each structure, the interfacial area was estimated using the PISA webserver (1-3) between the spike protein and each chain of the Fab. The total antibody footprint area was the sum of the heavy chain and light chain interfacial areas. The mean and standard error of the footprint areas were then calculated. Note that we only sought a rough estimate of the footprint area in order to set a threshold on the conservation fraction.

References

1. Krissinel E, Henrick K. Detection of Protein Assemblies in Crystals. In: R. Berthold M, Glen RC, Diederichs K, Kohlbacher O, Fischer I, editors. Computational Life Sciences. 3695. Berlin, Heidelberg: Springer Berlin Heidelberg; 2005. p. 163-74.

2. Krissinel E, Henrick K. Inference of Macromolecular Assemblies from Crystalline State. Journal of Molecular Biology. 2007;372(3):774-97.

3. Krissinel E. Crystal contacts as nature's docking solutions. Journal of Computational Chemistry. 2010;31(1):133-43.
