# Supplementary material for: Design of immunogens for eliciting antibody responses that may protect against SARS-CoV-2 variants
Source: PLoS Comput Biol. 2022 Sep 26;18(9):e1010563. doi: 10.1371/journal.pcbi.1010563 (PMC9536555; doi:10.1371/journal.pcbi.1010563)
Supplement: S3 Text — (DOCX) [file pcbi.1010563.s006.docx]

## S3 Text. Collection and processing of GISAID SARS-CoV-2 sequences

SARS-CoV-2 genomes with collection dates between December 30, 2019 and July 27, 2021 were downloaded from the GISAID database (~3.3M total). Each genome was translated into its amino acid sequence in all three reading frames using Biopython version 1.78 (1). The proper reading frame for each genome was considered to be the one that contains the subsequence MFVFLVLLP, the first 9 amino acids of the spike protein. If none of the reading frames contained this subsequence, then the genome was discarded. The end of the spike protein was found by identifying the next stop codon after the subsequence. Using the beginning and end of the spike protein thus identified, the sequence of the spike protein was extracted. As a quality check, spike protein sequences were discarded if they were not between 1270 and 1273 amino acids long, or if they contained 3 or more ambiguous amino acids, leading to ~300,000 high quality sequences. These sequences were then aligned with the Wuhan reference sequence (NCBI: NC_045512.2) using Clustal Omega version 1.2.4 (2) with default settings (Clustal Omega was used instead of ClustalW because the number of sequences was very large), and alignment gaps in the Wuhan reference sequence were removed to produce the final alignment. Sequences are not provided due to GISAID terms of service.

References

1. Cock PJA, Antao T, Chang JT, Chapman BA, Cox CJ, Dalke A, et al. Biopython: freely available Python tools for computational molecular biology and bioinformatics. Bioinformatics. 2009;25(11):1422-3.

2. Sievers F, Wilm A, Dineen D, Gibson TJ, Karplus K, Li W, et al. Fast, scalable generation of high-quality protein multiple sequence alignments using Clustal Omega. Mol Syst Biol. 2011;7:539.
