# Supplementary material for: Design of immunogens for eliciting antibody responses that may protect against SARS-CoV-2 variants
Source: PLoS Comput Biol. 2022 Sep 26;18(9):e1010563. doi: 10.1371/journal.pcbi.1010563 (PMC9536555; doi:10.1371/journal.pcbi.1010563)
Supplement: S4 Text — (DOCX) [file pcbi.1010563.s007.docx]

## S4 Text. Choice of number of escape mutations considered

We chose the top 34 escape mutations because mutating too many residues was likely to destabilize the RBD. First, we noted that the deep mutational scanning data was based on RBDs with 1-7 mutations, so we could not design RBDs with more than 7 mutations. Furthermore, rather than mutating the maximum number of 7 residues, we chose to mutate 5 residues per RBD to further decrease the probability of destabilizing the RBD. Since the mutations were divided into 2 groups, mutating 5 residues per RBD allowed 10 unique residues to be mutated in total. The top 34 escape mutations was the largest set that contained 10 unique residues (the top 35 escape mutations contained 11 unique residues).

If 7 mutations per RBD were allowed, this corresponded to 14 unique residues. The top 40 escape mutations contained 14 unique residues. However, the total escape of the top 40 escape mutations was only moderately larger than the total escape of the top 34 escape mutations, so choosing the top 34 mutations did not significantly affect the escape potential of our antigens.
