# Supplementary material for: Design of immunogens for eliciting antibody responses that may protect against SARS-CoV-2 variants
Source: PLoS Comput Biol. 2022 Sep 26;18(9):e1010563. doi: 10.1371/journal.pcbi.1010563 (PMC9536555; doi:10.1371/journal.pcbi.1010563)
Supplement: S5 Text — (DOCX) [file pcbi.1010563.s008.docx]

## S5 Text. Titers do not depend on the number of variant antigens encountered in each B cell - FDC interaction

We also simulated the case in which antigens are heterogeneously distributed on the FDC, so a B cell encounters only one randomly chosen antigen in each cycle. In S12 Fig, the mean panel titers are not significantly different from the all-antigen case. The main difference between the all-antigen and one-antigen case is that the optimal concentration is lower for the all-antigen case (S13 Fig). An optimal concentration exists because low concentrations induce GC collapse while high concentrations are incapable of discriminating between low-affinity and high-affinity clones.

In the one-antigen case, a B cell that has high affinity to a particular antigen will likely survive a cycle if it encounters that antigen on the FDC, but it will likely die if it encounters a different antigen for which the affinity is below a threshold. Over many cycles, the B cell is likely to encounter a different antigen at some point and die. Increasing the concentration counteracts this because a higher concentration will decrease the probability of low-affinity B cells dying. Thus, the optimal concentration is higher for the one-antigen case. It is important to note that our goal in this study is not to design antigens and immunization schemes that result in bnAbs that focus their binding footprint only on the conserved residues and can protect against a large set of variants. Having a high concentration of antigen in the one-antigen case would result in a polyclonal response of strain-specific antibodies, just like the all-antigen case, not bnAbs. Our goal in this study is to elicit a polyclonal antibody response that can protect against a limited set of variants. For this situation, manipulating antigen concentration can mitigate the difference between heterogeneous and homogeneous distribution of antigens on FDCs.
