# Supplementary figures and images for: Design of immunogens for eliciting antibody responses that may protect against SARS-CoV-2 variants
Source: PLoS Comput Biol. 2022 Sep 26;18(9):e1010563. doi: 10.1371/journal.pcbi.1010563 (PMC9536555; doi:10.1371/journal.pcbi.1010563)

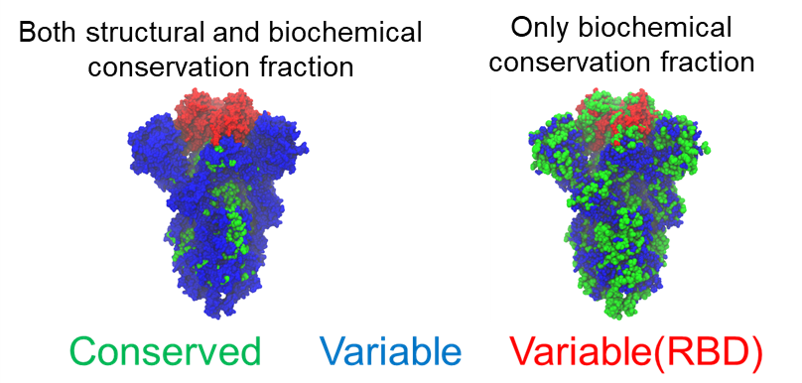

Supplement: S1 Fig — Green residues have conservation fractions above 0.8, blue residues have conservation fractions below 0.8 and are not in the RBD, and red residues have conservation fractions below 0.8 and are in the RBD. (TIF) [file pcbi.1010563.s009.tif]

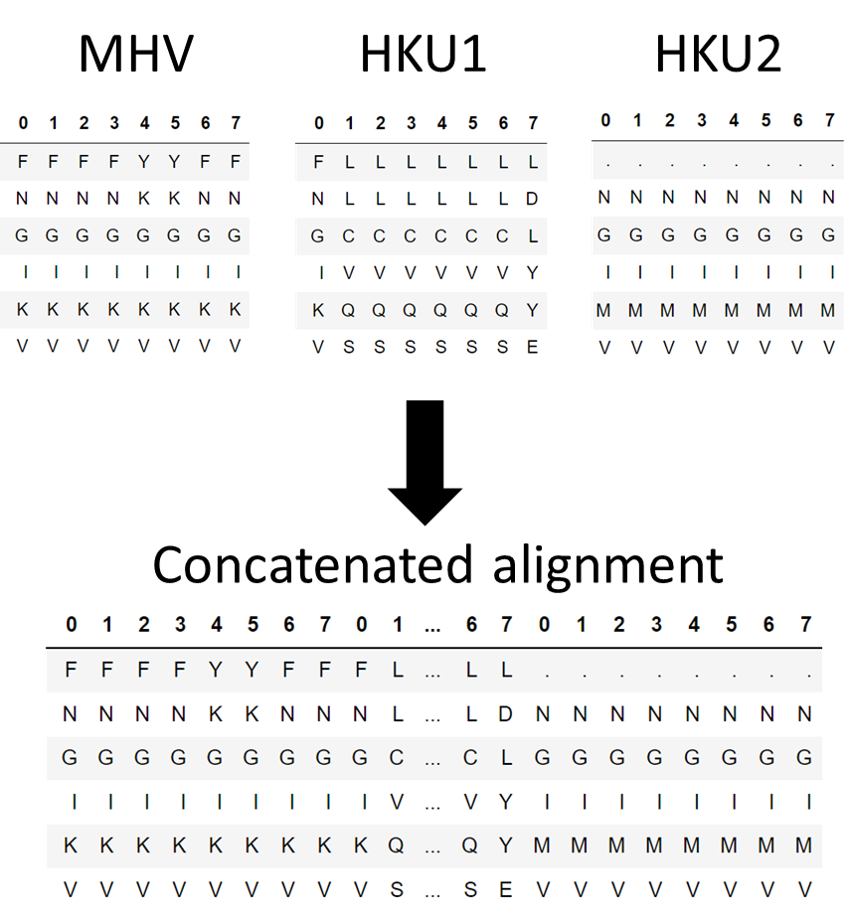

Supplement: S2 Fig — The number of coronaviruses and size of the alignments have been reduced for clarity and to match the example table from Fig 1. (TIF) [file pcbi.1010563.s010.tif]

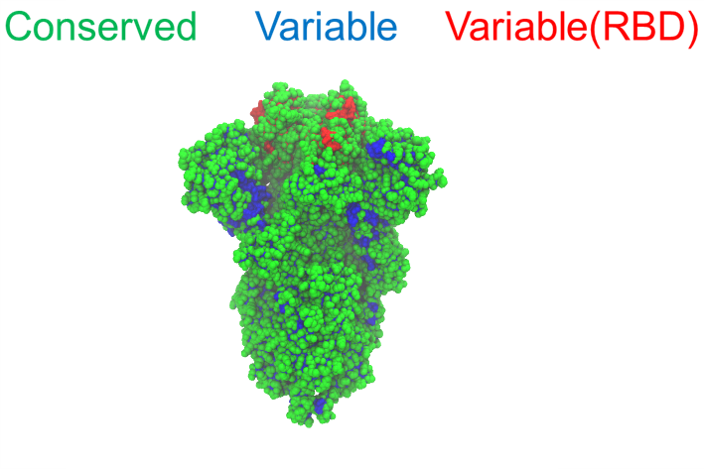

Supplement: S3 Fig — Green residues have conservation fractions above 0.8, blue residues have conservation fractions below 0.8 and are not in the RBD, and red residues have conservation fractions below 0.8 and are in the RBD. PDB structures used to calculate the structural conservation fraction include the WT structure (PDB ID: 6VXX), the Alpha variant structure (PDB ID: 7LWI), the Beta variant structure (PDB ID: 7LWS), the Delta variant structure (PDB ID: 7V7Q), and the Gamma variant structure (PDB ID: 7M8K). ~300,000 spike sequences (obtained from GISAID) were used to calculate the biochemical conservation fraction. (TIF) [file pcbi.1010563.s011.tif]

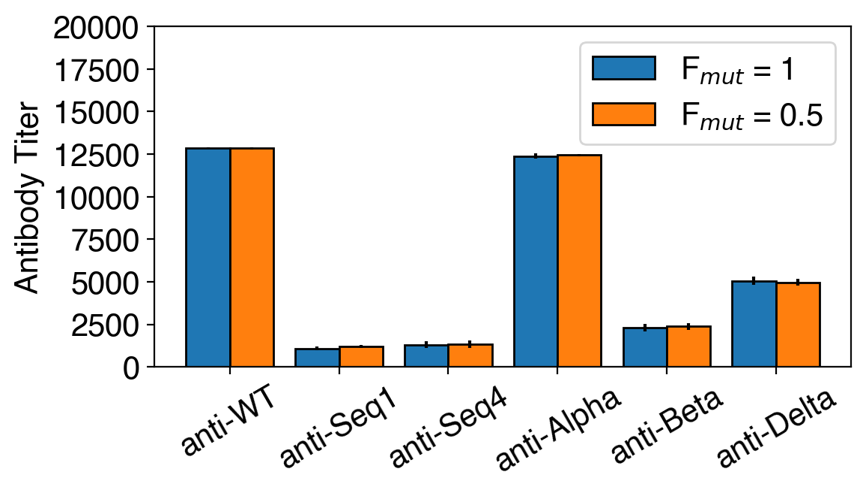

Supplement: S4 Fig — Sequences 2 and 3 are similar to sequence 1, so they are omitted for clarity. Sequences 5 and 6 are also omitted because they are similar to sequence 4. Fmut is the scaling factor for mutated residues. (TIF) [file pcbi.1010563.s012.tif]

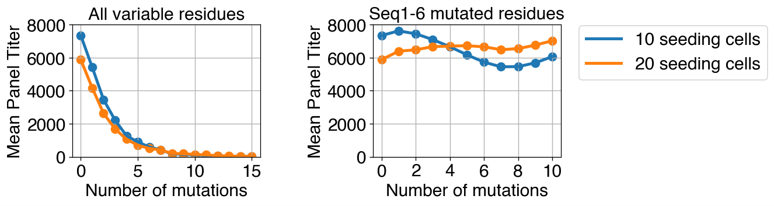

Supplement: S5 Fig — Mutations occur either in any variable residues (All variable residues) or in the same residues that are mutated in the sequences 1–6 (Seq1-6 mutated residues). B cells are assumed to encounter all antigens at a time on the FDC. (TIF) [file pcbi.1010563.s013.tif]

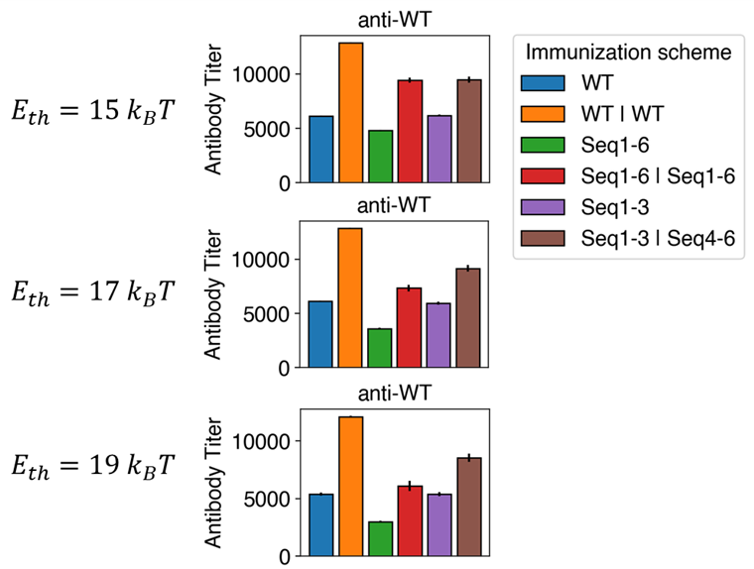

Supplement: S6 Fig — (TIF) [file pcbi.1010563.s014.tif]

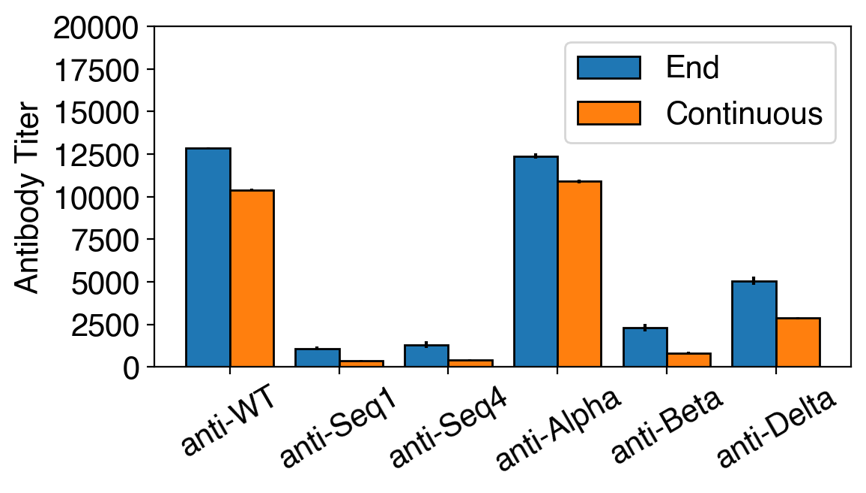

Supplement: S7 Fig — Sequences 2 and 3 are similar to sequence 1, so they are omitted for clarity. Sequences 5 and 6 are also omitted because they are similar to sequence 4. “End” indicates that the antibody titer is calculated using the B cell population at the end of the simulation, and “Continuous” indicates that the titer is calculated using the population of B cells that continuously exit the GC as memory or plasma cells. (TIF) [file pcbi.1010563.s015.tif]

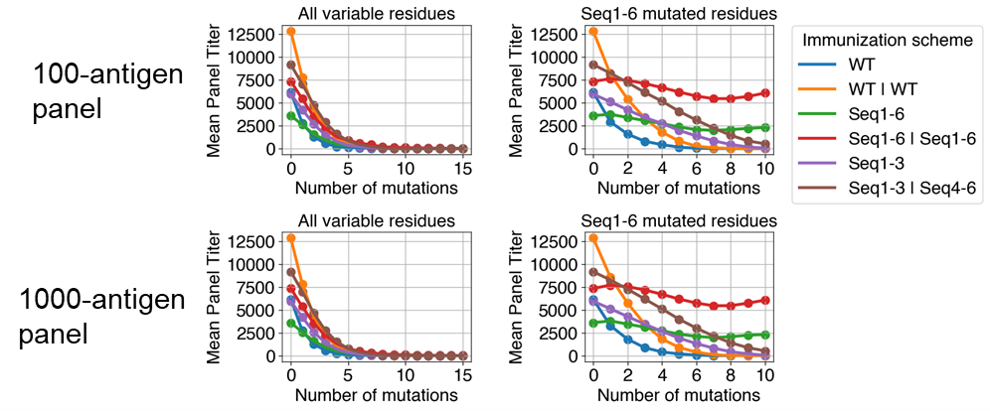

Supplement: S8 Fig — Mutations occur either in any variable residues (All variable residues) or in the same residues that are mutated in the sequences 1–6 (Seq1-6 mutated residues). Panel titers are calculated against panels of 100 antigens and 1000 antigens. Mutated residues take on a value of -4. B cells are assumed to encounter all antigens at a time on the FDC. (TIF) [file pcbi.1010563.s016.tif]

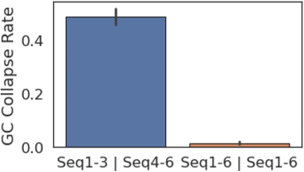

Supplement: S9 Fig — (TIF) [file pcbi.1010563.s017.tif]

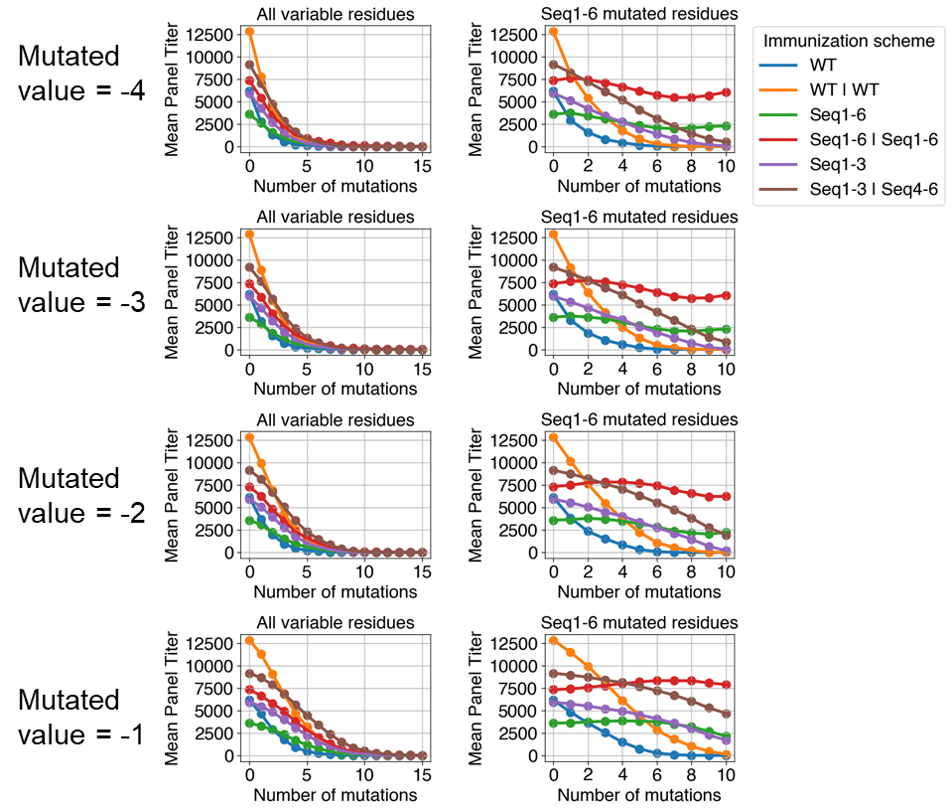

Supplement: S10 Fig — Mutations occur either in any variable residues (All variable residues) or in the same residues that are mutated in the sequences 1–6 (Seq1-6 mutated residues). B cells are assumed to encounter all antigens at a time on the FDC. Mutated residues take on values of -4, -3, -2, or -1, as indicated. (TIF) [file pcbi.1010563.s018.tif]

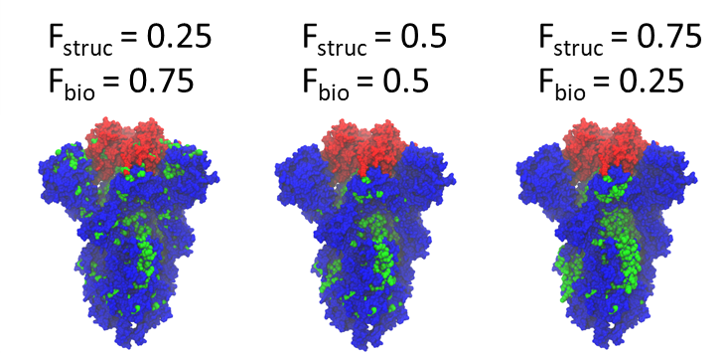

Supplement: S11 Fig — Green residues have conservation fractions above 0.8, blue residues have conservation fractions below 0.8 and are not in the RBD, and red residues have conservation fractions below 0.8 and are in the RBD. (TIF) [file pcbi.1010563.s019.tif]

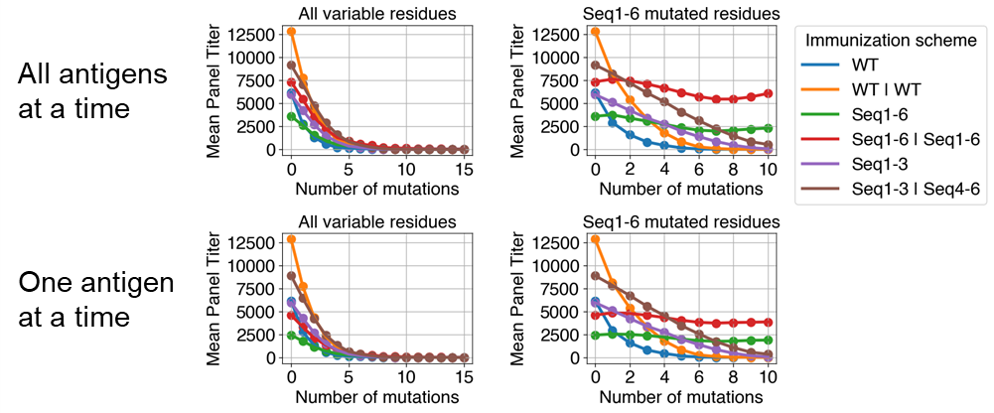

Supplement: S12 Fig — Mutations occur either in any variable residues (All variable residues) or in the same residues that are mutated in the sequences 1–6 (Seq1-6 mutated residues). Mutated residues take on values of -4. B cells encounter either all antigens at a time (All-antigen) or one antigen at a time (One-antigen) on the FDC. (TIF) [file pcbi.1010563.s020.tif]

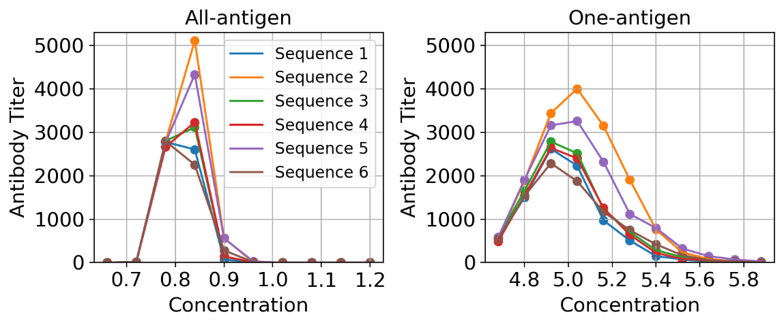

Supplement: S13 Fig — (TIF) [file pcbi.1010563.s021.tif]
